# Obligatory Role for Endothelial Heparan Sulphate Proteoglycans and Caveolae Internalization in Catestatin-Dependent eNOS Activation

**DOI:** 10.1155/2014/783623

**Published:** 2014-07-20

**Authors:** Sara Fornero, Eleonora Bassino, Roberta Ramella, Clara Gallina, Sushil K. Mahata, Bruno Tota, Renzo Levi, Giuseppe Alloatti, Maria Pia Gallo

**Affiliations:** ^1^Department of Life Sciences and Systems Biology, University of Turin, Via Accademia Albertina 13, 10123 Turin, Italy; ^2^Veterans Affairs San Diego Healthcare System, 3350 La Jolla Village Drive, San Diego, CA 92161, USA; ^3^Department of Medicine, University of California, 9500-0838 Gilman Drive, La Jolla, San Diego, CA 92093, USA; ^4^DIBEST Department, University of Calabria, 87030 Arcavacata di Rende, Italy; ^5^National Institute for Cardiovascular Research, Via Irnerio 48, 40126 Bologna, Italy

## Abstract

The chromogranin-A peptide catestatin modulates a wide range of processes, such as cardiovascular functions, innate immunity, inflammation, and metabolism. We recently found that the cardiac antiadrenergic action of catestatin requires a PI3K-dependent NO release from endothelial cells, although the receptor involved is yet to be identified. In the present work, based on the cationic properties of catestatin, we tested the hypothesis of its interaction with membrane heparan sulphate proteoglycans, resulting in the activation of a caveolae-dependent endocytosis. Experiments were performed on bovine aortic endothelial cells. Endocytotic vesicles trafficking was quantified by confocal microscopy using a water-soluble membrane dye; catestatin colocalization with heparan sulphate proteoglycans and caveolin 1 internalization were studied by fluorimetric measurements in live cells. Modulation of the catestatin-dependent eNOS activation was assessed by immunofluorescence and immunoblot analysis. Our results demonstrate that catestatin (5 nM) colocalizes with heparan sulphate proteoglycans and induces a remarkable increase in the caveolae-dependent endocytosis and caveolin 1 internalization, which were significantly reduced by both heparinase and wortmannin. Moreover, catestatin was unable to induce Ser^1179^ eNOS phosphorylation after pretreatments with heparinase and methyl-*β*-cyclodextrin. Taken together, these results highlight the obligatory role for proteoglycans and caveolae internalization in the catestatin-dependent eNOS activation in endothelial cells.

## 1. Introduction

Chromogranin A (CgA) is a 48 kDa acidic glycoprotein [1–3] and the main component of granins, a family of proteins abundantly expressed in large dense core vesicles of neuroendocrine cells, neurons, and other secreting cells including cardiac cells [4]. In the heart CgA is costored and cosecreted, respectively, with catecholamines and natriuretic peptides [4]. Catestatin (CST: hCgA_352-372_) is a 21 amino acid cationic and hydrophobic peptide derived from the proteolytic cleavage of CgA. It was firstly discovered as an endogenous allosteric nicotinic-cholinergic antagonist [5], but it is now established as a multifunctional peptide modulating several organs/systems, including the cardiovascular system [6, 7]. In particular, it has been shown that CST administration induces vasodilation by multiple mechanisms. One mechanism relies on the CST-dependent inhibition of catecholamine secretion, through noncompetitive binding at the nicotinic cholinergic receptor [5, 8]. Another potential mechanism for the CST-induced vasodilation is through histamine release [9, 10], which has been shown* in vitro *in mast cells mimicking the receptor-independent peptidergic pathway proposed for mastoparan and for other cationic and amphipathic peptides [10]. Finally, CST has been shown to decrease sympathetic vascular tone by a direct excitatory effect on the GABAergic inhibitory neurons of the caudal ventrolateral medulla (CVLM), resulting in decreased sympathetic drive and a subsequent fall in arterial pressure and heart rate [11]. Therefore, CST plays crucial roles in the regulation and development of hypertension. In humans, CST plasma levels are decreased not only in hypertensive patients but also in their still-normotensive offsprings [12]. However, plasma CST level has recently been shown to be elevated in patients with heart failure [13] or with coronary heart diseases [14].

Several studies indicate that exogenous CST rescues hypertension [15] and improves baroreflex sensitivity [16] and heart rate variability [17] in CgA knockout mice.

In addition to its important role in the control of blood pressure, CST is now emerging as a peptide that has direct cardiovascular actions under both basal and stimulated conditions, suggesting that CST-induced negative inotropism and lusitropism may be important components of its hypotensive action [6, 18].

In particular in a previous study we showed a PI3K-dependent nitric oxide (NO) release induced by CST in endothelial cells [18], suggesting one of the intracellular mechanisms underlying the cardiac antiadrenergic action of this peptide.

Based on these findings, the aim of the present study was to look into the initiating step required for this intracellular cascade, as up to now, high affinity membrane receptors for CST remain unknown. We therefore tested the hypothesis of a receptor-independent cell membrane interaction.

This hypothesis was supported by several evidences: CST adopts a *β*-sheet structure when interacting with negatively charged membranes and may thus directly pass through cell plasma membrane [19], and this property is believed to be responsible for the antimicrobial activity against a wide array of skin pathogens, including bacteria, yeast, and fungi [20, 21]. Moreover, in endothelial cells the CgA-derived peptide vasostatin-1 (VS-1), bearing cationic and amphipathic properties, interacts with cell-surface proteoglycans and activates eNOS phosphorylation on Ser^1179^ residue through a PI3K-dependent endocytosis-coupled mechanism [22, 23]. The above findings prompted us to test whether CST, by virtue of its cationic and amphipathic properties, can work like VS-1.

## 2. Materials and Methods

### 2.1. Cell Culture, Solutions, and Drugs

Bovine Aortic Endothelial (BAE-1) cells (European Collection of Cell Cultures, Salisbury, Wiltshire, UK) were maintained in Dulbecco's Modified Eagle's medium (DMEM, Sigma, St. Louis, MO, USA) added with 10% heat-inactivated fetal calf serum (FCS, Biowhittaker, Verviers, Belgium, lot 1SB0019), 100 U/mL penicillin, 100 *μ*g/mL streptomycin, and 2 mM glutamine, at 37°C, 5% CO_2_. Cells were used at passages 2–6 and maintained in 1% FCS 24 h before the experiments. M*β*CD, H:ase, and Wm were purchased from Sigma.

Tyrode's standard solution used for cell washes in colocalization experiments contained (mM) 154 NaCl, 4 KCl, 2 CaCl_2_, 1 MgCl_2_, 5.5 D-glucose, 5 Hepes, pH adjusted to 7.34 with NaOH.

CST was a kind gift by Professor Mahata and was synthesized by the solid-phase method, using 9-fluorenylmethoxycarbonyl protection chemistry [24]. The concentration of CST tested on BAE-1 cells was 5 nM, in the range of the circulating levels of CgA (1.36 nM) found in healthy humans [12].

Cy3-CST was purchased from Phoenix Pharmaceuticals.

### 2.2. Antibodies

The expressions of total eNOS and *β*-actin were detected with monoclonal antibodies (Invitrogen and BD Biosciences, resp.), while P^Ser1179^eNOS and Caveolin 1 (Cav1) were evidenced with polyclonal antibodies that were purchased, respectively, from Invitrogen and Sigma. heparan sulphate was stained with a mouse monoclonal antibody (MAB2040, Millipore) that we labeled with Alexa Fluor 488 using the APEX Antibody Labeling Kit (Invitrogen).

The secondary antibodies employed for immunofluorescence experiments were Alexa Fluor 488 anti-mouse (Molecular Probes) for total eNOS and Cy3 anti-rabbit (Sigma) for P^Ser1179^eNOS and Cav1. For Western blot experiments we used horseradish peroxidase-conjugated secondary antibodies: anti-mouse for *β*-actin (Invitrogen) and anti-rabbit for P^Ser1179^eNOS (Amersham).

### 2.3. Immunofluorescence and Confocal Microscopy

Cells grown on cover slides were fixed for 20 minutes in 4% paraformaldehyde in 0.1 M phosphate buffer (PB), pH 7.3. After three washes with Dulbecco's phosphate buffer saline (PBS), cells were incubated 20 minutes with 0.3% Triton and 1% bovine serum albumin (BSA, Sigma) in PBS and stained with the primary antibody 24 h at 4°C. Cover slides were washed twice with PBS and incubated 1 h at room temperature with the secondary antibody. After two washes in PBS cover slides were mounted on standard slides with DABCO (Sigma) and observed after 24 h under confocal microscope. Fluorimetric measurements were also performed with confocal microscopy, using an Olympus Fluoview 200 laser scanning confocal system (Olympus America Inc., Melville, NY, USA) mounted on an inverted IX70 Olympus microscope, equipped with a 60X Uplan Fl (NA 1.25) and a 100X Uplan Fl (NA 1.3) oil-immersion objectives. Image processing and analysis were performed with ImageJ software (Rasband, W.S., U.S. National Institutes of Health, Bethesda, MA, http://rsb.info.nih.gov/ij/, 1997–2013).

### 2.4. Western Blot Analysis

BAE-1 cells were lysed with lysis buffer (100 mM Tris HCl, pH 8.0, 1 mM MgCl_2_, plus inhibitor cocktail) and incubated at −80°C overnight. An equal volume of sucrose buffer containing 20 mM Tris Hepes pH 7.4 and 315 mM sucrose plus inhibitor cocktail was added and cell lysate was forced throughout a 25-gauge needle attached on a 1 mL syringe for several times. The inhibitor cocktail contained 2 *μ*g/mL aprotinin, 0.1 mM PMSF, 1 mM sodium orthovanadate, and 20 mM sodium fluoride. Protein lysates (15 *μ*g of protein per lane) were run on 8% gradient SDS-PAGE gel, transferred to a polyvinylidene fluoride membrane (PVDF; Millipore), and blocked overnight in TBST (10 mM Tris-HCl pH 7.5, 0.1 M NaCl, and 0.1% Tween 20) plus 5% nonfat dry milk (Biorad). PVDF was incubated, with gentle agitation, 1 h at 30°C with a polyclonal anti-P^Ser1179^eNOS antibody. Membranes were washed three times with TBST and were incubated 1 h at room temperature with horseradish peroxidase-conjugated secondary antibodies before being washed again three times with TBST. Protein band detection was performed by chemiluminescence using the Super Signal West Pico Kit (Pierce).

### 2.5. Endocytotic Vesicles Trafficking

The water-soluble membrane dye N-(3-triethylaminopropyl)-4-(p-dibutylaminostyryl) pyridinium dibromide (FM 1-43, Invitrogen) was used to label plasmalemma-derived vesicles, as previously described [25], and therefore to quantify endocytosis by confocal microscopy. BAE-1 cells grown on glass-bottom dishes (MatTek Corporation) were incubated at 37°C-5% CO_2_ for 15 min in PBS containing 5 *μ*g/mL FM 1-43, for control condition and in Hepes buffered saline solution (HBSS) plus 5 nM CST for experimental condition. Before fixation for 20 minutes in 4% paraformaldehyde, cells were washed three times in ice-cold dye-free PBS containing BSA (6 mg/mL, fraction *V*, 99% pure, endotoxin free; Sigma) to remove all unincorporated fluorescent probes from the external surface of endothelial cells. To investigate the involvement of heparan sulphate proteoglycans (HSPGs) in CST-dependent processes, we pretreated BAE-1 cells with 2 U/mL Heparinase III (H:ase) from* Flavobacterium heparinum* (Sigma) for 3 h at 37°C, 5% CO_2_. After two washes with PBS, cells were incubated for 15 min with PBS plus 5 nM CST and we proceeded as described above. To investigate the role of PI3K pathway in the formation of vesicles, we pretreated BAE-1 cells with the PI3K inhibitor Wortmannin (Wm, 100 nM) for 20 min and then we added 5 nM CST. For each experiment we randomly acquired three fields/sample. Endocytosis quantification was performed with ImageJ software: briefly, after creating the *z*-axis reconstruction (i.e., average of the slices) of the stack, the quantity of intracellular stained vesicles was analyzed by evaluating the fluorescence intensity/cell/field.

### 2.6. Colocalization Studies with CST and HSPGs

Monoclonal anti-heparin/heparan sulfate antibody (anti-HSPGs MAB2040, Millipore) was conjugated with Alexa Fluor 488 using a commercial kit (APEX Antibody Labeling Kit, Invitrogen); briefly 10 *μ*L of Alexa Fluor-anti-HSPGs was freshly prepared before the experiment. Fluorescent CST (Cy3-CST) was purchased from Phoenix Pharmaceuticals. BAE-1 cells grown on 35 mm glass bottom dishes were treated at 4°C for 10 min with Alexa Fluor-anti-HSPGs, followed by a rapid (2 min) exposure to Cy3-CST (5 nM), and after two washes in Tyrode's solution cells were rapidly transferred to the stage of the confocal microscope. Single fields were captured (60x objective) using separately 488 and 568 nm laser lines to avoid bleed through and ensure specific fluorescence. Images were analysed with the ImageJ/Fiji command “Colocalization Threshold.”

### 2.7. Transfection with GFP-Cav1

Transfection of BAE-1 cells was performed with the cationic liposome Lipofectamine (Lipofectamine 2000TM, Life Technologies, Carlsbad, CA). Endothelial cells were seeded into P-10 flasks (Costar, Cambridge, MA) at 30% confluence and allowed to attach and grow to reach 90% confluence; transfection was performed using 1 *μ*L of Lipofectamine 2000TM and 1 *μ*g of GFP-Cav1 plasmid DNA in 120 *μ*L of Opti-MEM (Life Technologies) for 6 h at 37°C, 5% CO_2_. Before observations, transfected BAE-1 cells were incubated 24 h to allow protein expression. Confocal fluorimetric measurements were performed as described in the “Immunofluorescence and Confocal Microscopy” section.

### 2.8. Statistical Analysis

All values are presented as the mean ± S.E.M. Statistical comparisons were performed with ANOVA analysis followed by Bonferroni correction for post hoc tests. Significance was accepted at a *P* level < 0.05.

## 3. Results 

### 3.1. CST Induces Endocytotic Vesicles Formation That Is Abolished by Both Heparinase and Wortmannin

The role of CST in the endocytotic process was evaluated by incubating BAE-1 cells with the styryl pyridinium membrane probe FM 1-43 (5 *μ*g/mL), in order to visualize plasmalemma-derived endocytotic vesicles.

To quantify these results we randomly acquired three fields per sample in each experiment and then evaluated the fluorescence intensity value/n° cell/field (see Section 2), in control condition and after CST stimulation.

**Figure 1:**
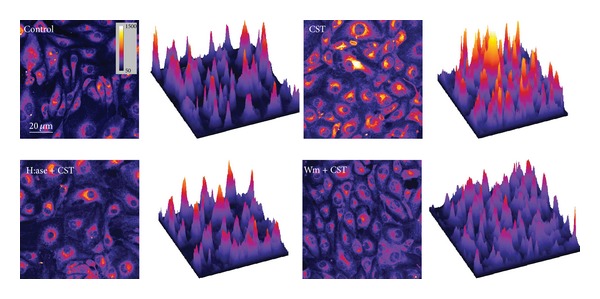
CST stimulates a PI3K-proteoglycan dependent endocytotic machinery in BAE-1 cells. BAE-1 cells incubated with the water-soluble styryl pyridinium membrane dye (FM 1-43). Pseudocolor images better show the fluorescence intensity increase correlated with the rise in vesicles formation consequent to stimulation with CST 5 nM. In CST + H:ase and CST + Wm samples fluorescence intensity was comparable to control levels. Surface plots close to each image display 3D graphs of pixels intensities in a pseudocolor image. The height and the color represent the pixel intensity.

We observed that CST (5 nM) induced a significant increase in the FM 1-43 fluorescence (Figure 1), indicating a stimulation of the endocytotic process.

As the molecular properties of CST (little, amphipathic, and cationic peptide) resemble those of the polycationic peptides (cell penetrating peptides or CPPs), we hypothesized that the first contact of this peptide with the cell surface takes place through proteoglycans, as we previously demonstrated for VS-1 [22]; internalization mechanisms mediated by HSPGs interaction involving different routes of endocytosis have indeed been described for several CPPs [26].

In order to verify this mechanism, HSPGs were selectively removed from the cell surface by pretreatment of cells with H:ase (2 U/mL). After this step, CST strongly reduced its ability to stimulate endocytosis (Figure 1), suggesting that CST binding to HSPGs is fundamental to start its intracellular cascade.

In a recent work we showed that in BAE-1 cells CST induced a PI3K-dependent NO release [18]. To investigate the role of PI3K pathway also in CST-dependent formation of endocytotic vesicles, we performed FM 1-43 detection in the presence of the PI3K inhibitor Wm (100 nM). As shown in Figure 1, pretreatment with Wm strongly reduced the CST-activated endocytosis.

**Figure 2:**
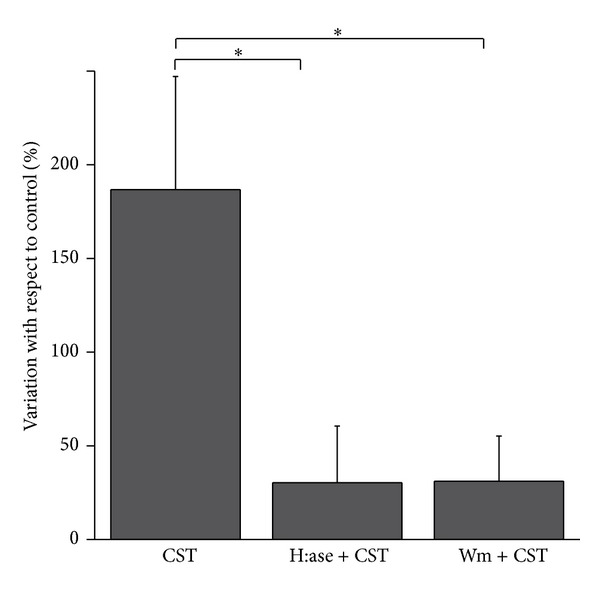
Endocytosis quantification. To quantify the endocytotic process stimulated by CST 5 nM, we evaluated FM 1-43 fluorescence intensity value/n° cell/field (see Section 2) in the different experimental conditions. The bar graph shows the variation of fluorescence intensity/n° cell/field with respect to control. CST = 186.8 ± 60.36%; H:ase + CST = 30.36 ± 30.24%; Wm + CST = 31.09 ± 24.11%; *n* = 7 endocytosis experiments (3 fields/sample, about 35 cells/fields); *P* < 0.05.

The bar graph in Figure 2 summarizes this set of experiments (percentage of fluorescence intensity/cell/field increase above control: CST = 186.8 ± 60.36%; H:ase + CST = 30.36 ± 30.24%; Wm + CST = 31.09 ± 24.11%; *n* = 7 endocytosis experiments: 3 fields/sample, about 35 cells/fields; *P* < 0.05).

### 3.2. CST Colocalizes with Heparan Sulphate Proteoglycans

To prove the interaction between CST and HSPGs we performed colocalization experiments in live cells by using Cy3-CST and Alexa Fluor 488-anti-HSPGs.

In these experiments the fluorescent antibody against heparan sulphate (1 : 200) was incubated 10 min at 4°C (to avoid endocytosis) with BAE-1 cells cultured on glass bottom dishes. The excess of antibody was removed by two washes and then cells were incubated 2 min at 4°C with 5 nM Cy3-CST. After two additional washes, cells were observed in confocal microscopy.

**Figure 3:**
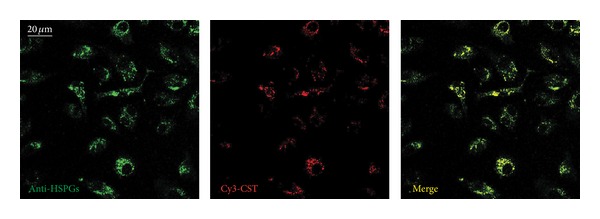
CST colocalizes with HSPGs. Representative confocal fluorescence images of BAE-1 cells incubated with both Alexa Fluor 488-anti-HSPGs (green signal) and Cy3-CST (red signal), showing the high levels of colocalization for CST and HSPGs (% of colocalization = 73.4 ± 0.02%, 3 separate experiments, 6 fields/sample, about 25 cells/field).

The results from these experiments (Figure 3) reveal that the colocalization between CST and HSPGs was very high (% of colocalization = 73.4 ± 0.02%, 3 separate experiments, 6 fields/sample, about 25 cells/field).

### 3.3. CST Induces Caveolin-1 Mobilization

As CST stimulates NO production [18] and several reports propose a mechanism of eNOS activation involving caveolae endocytosis [27, 28], we hypothesized that the endocytotic process triggered by CST was caveolae-dependent. To verify this hypothesis we followed the cellular localization of Cav1 by transfecting BAE-1 cells with GFP-Cav1. Cells were transfected with high efficiency (up to 95%) with GFP-Cav1 plasmid (see Section 2).

**Figure 4:**
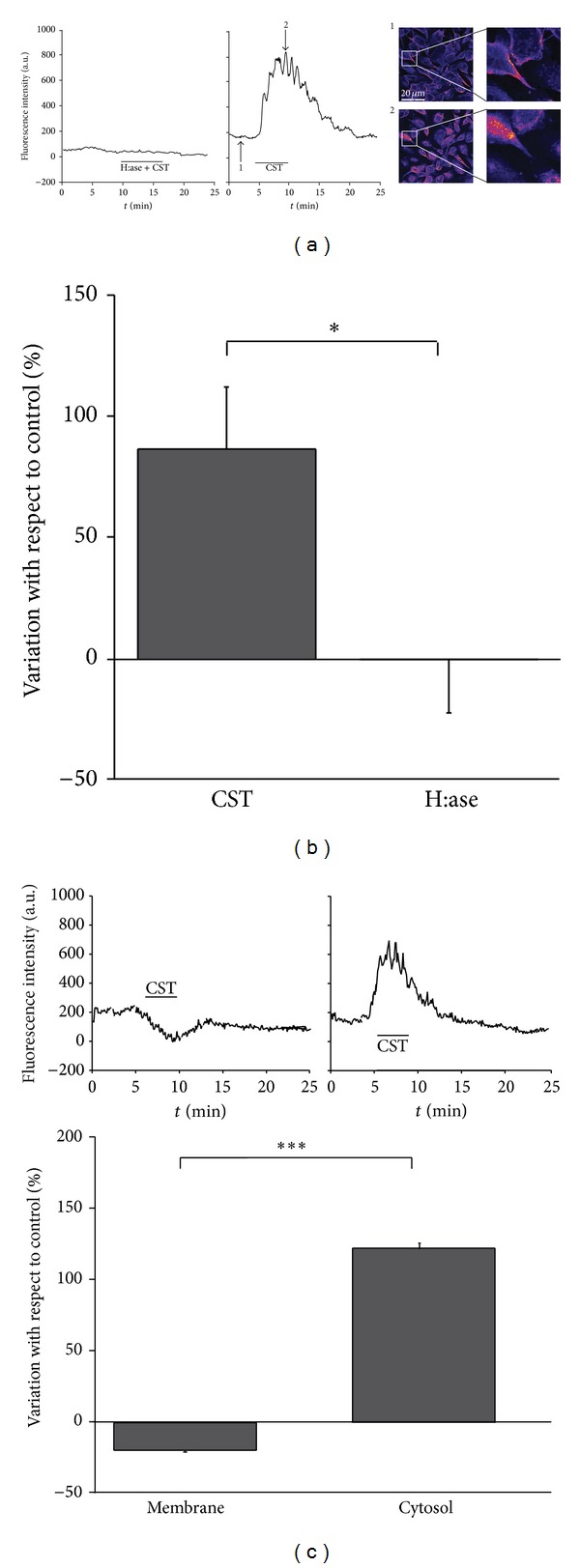
CST induces proteoglycan-dependent Cav1 internalization in GFP-Cav1 transfected BAE-1 cells. (a) Representative time course of the fluorescence intensity in single BAE-1 cells transfected with Cav-1-GFP and stimulated with H:ase + CST or with CST alone. Subpanels 1 and 2: confocal pseudocolor images of CST-treated BAE-1 cells from the correspondent time points indicated by the arrows, showing membrane (1) and cytosolic (2) localizations of GFP-Cav1 after CST treatment. (b) Bar graphs representing the percent variation with respect to control of the fluorescent GFP-Cav1 signal in the different experimental conditions (CST = 86.94 ± 25.51%; H:ase + CST = −0.07 ± 22.20%; *n* = 3 sets of experiments, 3 fields/sample, about 40 cells/fields; *P* < 0.05). (c) Bar graph representing the percent variation with respect to control of the fluorescent GFP-Cav1 signal during CST 5 nM treatment, respectively, in plasma membrane and in cytosol (membrane = −19.34 ± 1.22%; cytosol = 122.6 ± 3.49%; *n* = 17 cells; *P* < 0.05).

In transfected live cells GFP-Cav1 signal was confined in plasma membranes, while in the presence of CST 5 nM green fluorescence appeared clearly diffused in the cytosol, as a consequence of Cav1 internalization, thus producing a substantial increase of the overall fluorescent signal; pretreatment with H:ase strongly reduced CST ability to stimulate this process (Figures 4(a) and 4(b); percentage of fluorescence intensity variation above control: CST = 86.94 ± 25.51%; H:ase + CST = −0.07 ± 22.20%; *n* = 3 sets of experiments, 3 fields/sample, about 40 cells/fields; *P* < 0.05).

In order to further analyze the internalization of GFP-Cav1 in transfected BAE-1 cells, we evaluated GFP fluorescence values in specific regions localized in plasma membrane and in cytosol during CST 5 nM administration.

We found (Figure 4(c)) that during CST treatment GFP-Cav1 fluorescence intensity decreased in the plasma membrane while being increased in the cytosol (percentage of fluorescence intensity with respect to control: membrane= −19.34 ± 1.22%; cytosol = 122.60 ± 3.49%; *n* = 17 cells; *P* < 0.05). These results confirm that CST administration induces Cav1 displacement from plasma membrane; pretreatment of BAE-1 transfected cells with H:ase reverts CST effect (percentage of fluorescence intensity with respect to control: membrane = 54.27 ± 9.37%; cytosol = −98.46 ± 0.09%; *n* = 17 cells; *P* < 0.05; data not shown).

### 3.4. CST Breaks Caveolin-1/eNOS Colocalization

As CST stimulates endocytosis, induces Cav1 internalization, and, as shown in our previous report, enhances NO production [18] (Bassino et al., 2011), we hypothesized a mechanism for eNOS activation mediated by the displacement of the protein from Cav1 binding. Dissociation of eNOS from Cav1 has been indeed shown as a marker of eNOS activation [29].

**Figure 5:**
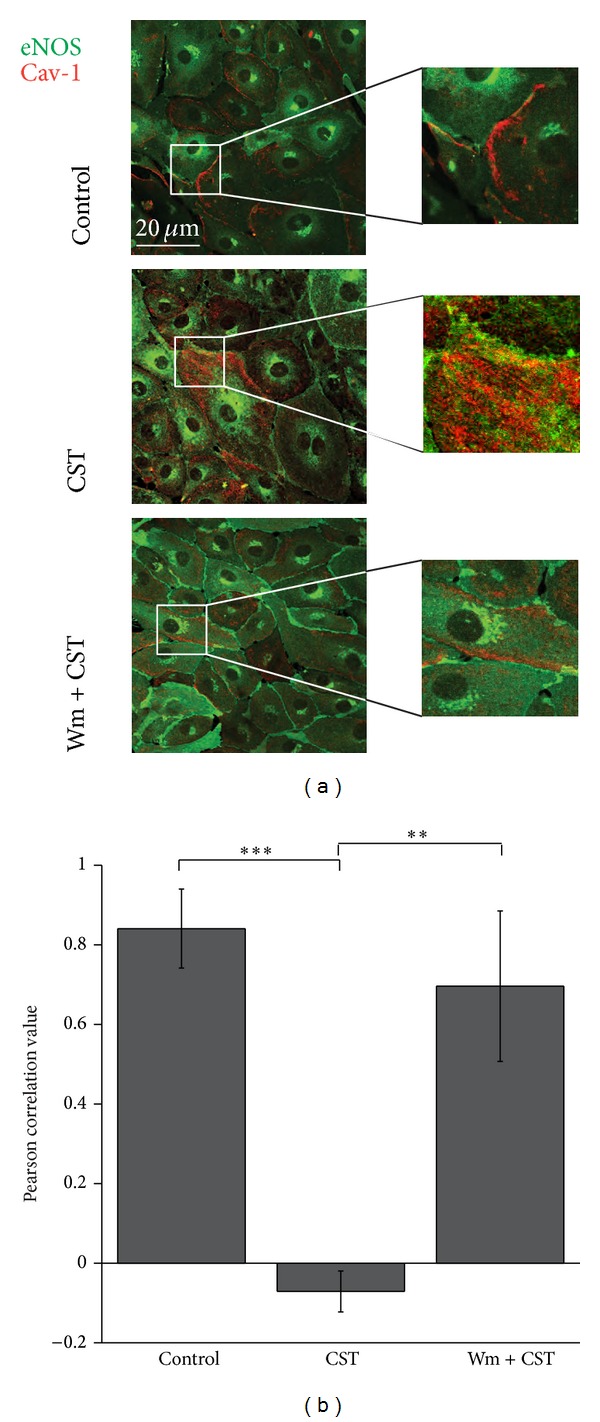
CST prevents Cav1/eNOS colocalization in a PI3K-dependent manner. (a) Confocal immunofluorescence images of BAE-1 cells showing the typical membrane localization of Cav1 (red staining) and eNOS (green staining). After CST administration, Cav1 dissociated from eNOS and staining diffused in the cytosol. This effect was reversed by Wm pretreatment. Immunofluorescence detection was carried out using Alexa Fluor 488 anti-mouse for total eNOS and Cy3 anti-rabbit for Cav1. Scale bar: 20 *μ*m. (b) Bar graph representing Pearson correlation values relative to Cav1 and eNOS staining in each experimental condition (control = 0.84 ± 0.09; CST = −0.07 ± 0.05; Wm + CST = 0.69 ± 0.18; *n* = 8 sets of experiments, 3 fields/sample, about 30 cells/fields; *P* < 0.05).

To verify this hypothesis we followed cellular colocalization of Cav1 and eNOS by immunofluorescence experiments (Figure 5). We observed that CST strongly reduced eNOS/Cav1 colocalization at plasma membrane detected in control condition. Moreover, Wm was able to restore this colocalization, confirming the role of PI3K in mediating CST intracellular signaling.

To quantify colocalization results, Pearson correlation coefficients were calculated for each experimental condition (Figure 5(b); control = 0.84 ± 0.09; CST = −0.07 ± 0.05; Wm + CST = 0.69 ± 0.18; *n* = 8 sets of experiments, 3 fields/sample, about 30 cells/fields; *P* < 0.05).

### 3.5. Caveolae Disruption and HSPGs Removal Both Abolish CST-Induced eNOS Phosphorylation

**Figure 6:**
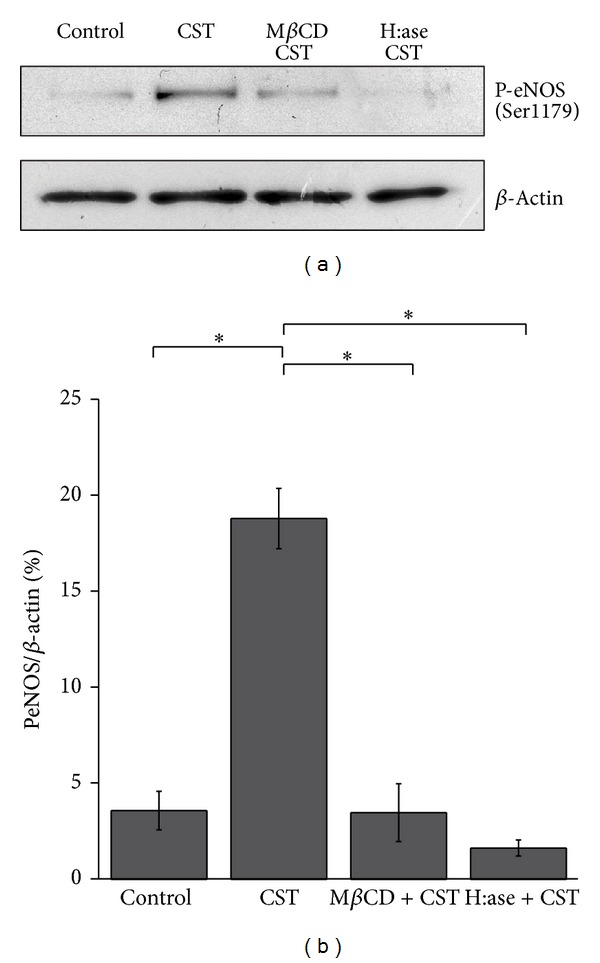
Both proteoglycans and caveolae are required to allow CST-dependent eNOS phosphorylation. (a) Typical Western blot experiment showing that CST-induced P^Ser1179^eNOS was reduced by both M*β*CD (5 mM, 30 min) and H:ase (2 U/mL). (b) P^Ser1179^eNOS/*β*-actin ratio of densitometric values from Western blots (%P^Ser1179^eNOS/*β*-actin: control = 3.59 ± 1; CST = 18.81 ± 1.57; M*β*CD + CST = 3.48 ± 1.5, H:ase + CST = 1.63 ± 0.41; *n* = 3; *P* < 0.05).

To strongly demonstrate the proposed pathway of a CST-dependent caveolae internalization and eNOS activation triggered by HSPGs, we evaluated the level of CST-induced P^Ser1179^eNOS after both caveolae disruption by methyl-*β*-cyclodextrin (MBCD, 5 mM) and HSPGs removal (by H:ase, 3 h). Our results from Western blot experiments (Figure 6) showed a significant reduction of the CST-dependent eNOS phosphorylation in both conditions (%P^Ser1179^eNOS/*β*-actin: control = 3.59 ± 1; CST = 18.81 ± 1.57; M*β*CD + CST = 3.48 ± 1.5, H:ase + CST = 1.63 ± 0.41; *n* = 3; *P* < 0.05), confirming the obligatory requirement of proteoglycans and caveolae integrity to allow the CST-dependent intracellular pathway activation.

## 4. Discussion

Our results show that in endothelial cells CST activates eNOS through HSPGs and caveolae-dependent endocytotic mechanism. The findings are consistent with our previous results showing that proteoglycans/PI3K-dependent caveolae endocytosis acts as the initiating factor for the intracellular cascade activated in endothelial cells by VS-1, the major N-terminal peptide derived from CgA [22, 23]. Our previous studies identifying a PI3K-dependent NO release from endothelial cells as the intracellular mechanisms involved in the cardiac antiadrenergic action of CST [18] prompted us to uncover the steps upstream the intracellular cascade activated by CST in endothelial cells.

First, we found that CST stimulates endocytotic vesicles formation (Figure 1), which corresponds with the biochemical reports on CST, reporting that this peptide, like other members of the CPPs family, exhibits membrane-interaction properties because of both its amphipathic structure and extended hydrophobic region. In particular, CD and NMR data indicate that CST folds into a short helical conformation that interacts with membranes and causes considerable disordering at the level of the phospholipid head groups. Moreover, two of the five residues of the helical region of CST are arginines, an amino acid that has been proposed to form hydrogen bond interactions with phospholipids [19].

It is widely accepted that some CPPs can directly translocate across the plasma membrane of cells. CPPs-mediated toxicity, manifested as a general increase in plasma membrane permeability, could reflect some of these observed features [30]. In this context, the ability of CST to target various microorganisms such as bacteria, fungi, and parasites [21] and also host cells such as neutrophils by a direct interaction with plasma membranes [31] falls in this general property of CPPs.

However, direct translocation has been observed only at high concentrations of CPPs (>10 *μ*M), while lower doses were shown to activate endocytotic mechanisms. For instance, studies of leukaemia cells showed that still at 2 *μ*M extracellular concentration R8-Alexa488 labeled vesicular structures, which were shown to be endosomes and lysosomes. Upon raising the concentration to 10 *μ*M, instead, the peptide was seen to flood into cells giving, in most cases, uniform labeling throughout the cytoplasm and nucleus [32]. In this perspective our results with CST 5 nM, higher than but comparable to the circulating concentrations of CgA found in healthy humans [12] (0.5–2 nM), are consistent with the activation of a more restricted endocytotic pathway.

Furthermore, our experiments also show that CST-activated endocytosis required the presence of HSPGs (Figures 1 and 2) on the surface of endothelial cells and that CST colocalizes with HSPGs (Figure 3). The strong anionic charge present in proteoglycans makes them favorable binding sites for cationic polymers, lipids, and polypeptides, which are used for drug and gene delivery [33, 34]. There are evidences that negatively charged carbohydrates, like HSPGs, located on the plasma membrane may serve as electrostatic traps for the cationic CPPs [30]. Interestingly, the most prominent glycosaminoglycans on the surface of endothelial cells are precisely heparan sulphates and one of the major protein core families of HSPGs is the membrane-bound glypicans, that are enriched in caveolae, where a series of molecules involved with eNOS signalling are localized [35, 36]. Furthermore, glypican-1 has been hypothesized to be the mechanosensor for eNOS phosphorylation and activation in the shear stress-induced response [37]. It could be speculated that the CST-mediated membrane perturbation through HSPGs binding and phospholipid interactions could resemble the acute membrane perturbation involved in shear stress.

This matter, together with our previous finding of CST-dependent eNOS activation [18], led us to propose a CST-induced mechanism of caveolae endocytosis and consequent eNOS activation. These assumptions are supported by our findings, from both Cav1 transfection and Cav1/eNOS immunofluorescence and colocalization experiments. Previous reports have proposed a mechanism of eNOS activation coupled with caveolae internalization [27, 28] and dissociation of eNOS from Cav1 has been shown as a marker of eNOS activation [29, 36].

In addition, we found that PI3K activity was required in both endocytosis and eNOS/Cav1 trafficking, thus representing the essential key for the CST-activated intracellular signaling. In agreement with these results, it is well known that PI3K/Akt mediated Ser^1179^ phosphorylation represents a common pathway among the multiple regulatory mechanisms affecting eNOS activity [36] and PI3K is widely reported to have an important role in membrane budding and fission in endothelial cells [38].

Finally with the last experiments (Figure 6) we confirmed our proposed pathway showing that caveolae disruption and HSPGs removal both abolished the CST-induced eNOS phosphorylation (Figure 6).

## 5. Conclusion

Based on our previous data on the CgA derived peptide VS-1 and the present findings, we hypothesize a novel signal transduction pathway for endogenous cationic and amphipathic peptides in endothelial cells: HSPGs interaction and caveolae endocytosis, coupled with a PI3K-dependent eNOS phosphorylation.

Moreover, giving the wide range of processes, such as innate immunity, inflammatory and autoimmune reactions, cardiovascular modulations, and several homeostatic regulations [39–45] affected by CST, the understanding of its physiological working represents a founding point for further applications.
